# Multiparametric MRI Analysis for the Identification of High Intensity Focused Ultrasound-Treated Tumor Tissue

**DOI:** 10.1371/journal.pone.0099936

**Published:** 2014-06-13

**Authors:** Stefanie J. C. G. Hectors, Igor Jacobs, Gustav J. Strijkers, Klaas Nicolay

**Affiliations:** 1 Biomedical NMR, Department of Biomedical Engineering, Eindhoven University of Technology, Eindhoven, The Netherlands; 2 Center for Imaging Research and Education (CIRE), Eindhoven, The Netherlands; 3 Biomedical Engineering and Physics, Academic Medical Center, University of Amsterdam, Amsterdam, The Netherlands; Wayne State University, United States of America

## Abstract

**Purpose:**

In this study endogenous magnetic resonance imaging (MRI) biomarkers for accurate segmentation of High Intensity Focused Ultrasound (HIFU)-treated tumor tissue and residual or recurring non-treated tumor tissue were identified.

**Methods:**

Multiparametric MRI, consisting of quantitative T_1_, T_2_, Apparent Diffusion Coefficient (ADC) and Magnetization Transfer Ratio (MTR) mapping, was performed in tumor-bearing mice before (n = 14), 1 h after (n = 14) and 72 h (n = 7) after HIFU treatment. A non-treated control group was included (n = 7). Cluster analysis using the Iterative Self Organizing Data Analysis (ISODATA) technique was performed on subsets of MRI parameters (feature vectors). The clusters resulting from the ISODATA segmentation were divided into a viable and non-viable class based on the fraction of pixels assigned to the clusters at the different experimental time points. ISODATA-derived non-viable tumor fractions were quantitatively compared to histology-derived non-viable tumor volume fractions.

**Results:**

The highest agreement between the ISODATA-derived and histology-derived non-viable tumor fractions was observed for feature vector {T_1_, T_2_, ADC}. R_1_ (1/T_1_), R_2_ (1/T_2_), ADC and MTR each were significantly increased in the ISODATA-defined non-viable tumor tissue at 1 h after HIFU treatment compared to viable, non-treated tumor tissue. R_1_, ADC and MTR were also significantly increased at 72 h after HIFU.

**Conclusions:**

This study demonstrates that non-viable, HIFU-treated tumor tissue can be distinguished from viable, non-treated tumor tissue using multiparametric MRI analysis. Clinical application of the presented methodology may allow for automated, accurate and objective evaluation of HIFU treatment.

## Introduction

Thermal ablation of tumors with High Intensity Focused Ultrasound (HIFU) [1], [2] is currently being introduced in the clinic for the treatment of both benign tumors, mainly uterine fibroids [3], [4], and malignant tumors, such as prostate [5], [6], [7] and breast tumors [8] and liver metastases [9], [10]. HIFU treatment of malignant tumors should cover the entire tumor, which requires adequate treatment planning, monitoring and evaluation. HIFU therapy is therefore commonly performed under image guidance, often using magnetic resonance imaging (MRI) [11], [12]. MRI facilitates treatment planning because of its excellent soft tissue contrast. Furthermore, MR thermometry allows for real-time temperature feedback during the procedure [12]. MRI is also well-suited for the evaluation of treatment outcome.

With MR thermometry, lethal thermal dose areas (*i.e.* tissue regions that received a thermal dose of at least 240 equivalent minutes (EM) at 43°C) can be identified [13]. However, a recent study on MR-guided HIFU treatment of a rabbit tumor model showed that the 240-EM thermal dose limit underestimates the necrotic tissue area immediately after HIFU treatment [14], possibly caused by the dependence of thermal dose necrosis thresholds on tissue type [13], [15].

For treatment evaluation commonly conventional MRI techniques are used, including T_2_-weighted and contrast-enhanced T_1_-weighted imaging. Kirkham *et al.* [7] reported a heterogeneous appearance of the tumor tissue on T_2_-weighted images up to 1 month after HIFU ablation of human prostate tumors, showing that T_2_-weighted imaging alone is inadequate for the assessment of necrosis. Furthermore, on contrast-enhanced T_1_-weighted imaging, an enhancing rim, surrounding the non-enhancing central core of necrosis, was observed, that can either originate from residual tumor tissue or from inflammation-induced hyperemia. A similar enhancement pattern was observed in other clinical studies on HIFU treatment of prostate tumors [6] and on radiofrequency (RF) ablation of kidney [16] and liver tumors [17]. Furthermore, the aforementioned study of HIFU treatment of a rabbit tumor model reported that contrast-enhanced T_1_-weighted imaging underestimates the area of necrosis in histology directly after HIFU treatment [14]. An additional drawback of contrast-enhanced T_1_-weighted imaging for the evaluation of HIFU treatment is the need for the injection of a Gadolinium (Gd) contrast agent. If immediate retreatment needs to be performed directly after treatment evaluation, the Gd contrast agent could interfere with the HIFU procedure. Presence of Gd in the tissue could induce susceptibility artifacts in the thermometry acquisitions, resulting in inaccurate temperature maps [18].

Several studies have reported on the evaluation of HIFU treatment with more advanced MRI protocols. In clinical studies, preliminary experiments were conducted in which diffusion-weighted imaging was used to evaluate HIFU treatment. A recent study on HIFU ablation of malignant liver lesions showed a significant increase in the Apparent Diffusion Coefficient (ADC) in the necrotic, HIFU-treated tumor tissue [19]. In contrast, a decrease in ADC was observed after HIFU treatment of uterine fibroids [20], [21]. The Magnetization Transfer Ratio (MTR) is another MRI parameter that has potential sensitivity for the distinction between HIFU-treated and non-treated tumor tissue. The MTR is a measure for the level of magnetization exchange between water protons and semi-solid macromolecular protons in tissue [22]. An increase in tissue MTR has been observed after thermal treatment of *ex vivo* porcine muscle tissue [23].

Overall, multiple studies have been published in which different MRI parameters for HIFU treatment evaluation were proposed. However, no quantitative studies on the correlation between changes in the different MRI parameters and histological analysis of the HIFU-treated lesion have been reported. Recently, multiparametric MRI has been proposed as a possibly suitable approach for the evaluation of HIFU treatment of prostate tumors [24]. To the best of our knowledge, multiparametric MR analysis consisting of quantitative assessment of HIFU-induced changes in the tumor tissue based on different combinations of MRI parameters has not yet been performed.

Therefore, the goal of the present study was to identify endogenous MRI biomarkers that can be used to distinguish between HIFU-treated and non-treated tumor tissue, using multiparametric MRI analysis combined with quantitative histological evaluation. Specifically, the multiparametric MRI protocol consisted of quantitative assessment of T_1_, T_2_, ADC and MTR and was used to assess changes in tumor tissue status as induced by HIFU treatment in a murine tumor model. The HIFU treatment consisted of partial ablation of the tumors to allow for internal reference between HIFU-treated and residual non-treated tumor tissue. MR evaluation of the tumor tissue was performed before and at 1 h and at 72 h after HIFU. The Iterative Self Organizing Data Analysis (ISODATA) clustering algorithm [25] was implemented and employed to segment the multispectral data into tissue populations with similar MRI parameter values. Cluster analysis was performed on different subsets of MRI parameters. The optimal set of MR parameters for the segmentation of HIFU-treated and non-treated tissue was determined by quantitative comparison between ISODATA-derived and histology-derived non-viable tumor volume fractions.

## Methods

### Ethics Statement

All animal experiments were performed according to the Directive 2010/63/EU of the European Parliament and approved by the Animal Care and Use Committee of Maastricht University (protocol: 2010-097).

### Murine tumor model

CT26.WT murine colon carcinoma cells (American Type Culture Collection (ATCC; CRL-2638)) were cultured as a monolayer at 37°C and 5% CO_2_ in RPMI-1640 medium (Invitrogen, Breda, The Netherlands), supplemented with 10% fetal bovine serum (Greiner Bio-One, Alphen a/d Rijn, The Netherlands) and 50 U/ml penicillin/streptomycin (Lonza Bioscience, Basel, Switzerland). Early passages (5–10) of the original ATCC batch were used for inoculation.

10–12 week-old Balb/c mice (Charles River, Maastricht, The Netherlands) were inoculated with 2×10^6^ CT26.WT cells subcutaneously in the right hind limb. Approximately 10 days after inoculation, tumors became palpable in all animals.

### Study design

Animals were subjected to MRI examination 24 h before (n = 14), 1 h after (n = 14) and 72 h after HIFU treatment (n = 7). A control group of non-treated animals (n = 7) was included. The time points of MRI examinations of the control animals were the same as for the HIFU-treated animals and are referred to as Day 0, Day 1 and Day 4. Directly after the last MRI experiment, the mice were sacrificed, and the tumors were dissected and processed for histological analysis. This study design led to three different groups for quantitative histology: animals sacrificed after the MRI examination at 1 h after HIFU treatment (n = 7, referred to as ‘1 h after HIFU’), animals sacrificed after the MRI examination at 72 h after HIFU treatment (n = 7, referred to as ‘72 h after HIFU’) and non-treated control animals (n = 7, referred to as ‘Control’).

### HIFU treatment

HIFU treatment was performed with the preclinical Therapy and Imaging Probe System (TIPS, Philips Research, Briarcliff Manor, NY, USA) [26], outside the MR system. Animals were initially anesthetized with 3% isoflurane in medical air and maintained with 1–2% isoflurane during HIFU treatment. Precautionary analgesia (buprenorphine, 0.1 mg/kg s.c.) was administered 30 min before treatment. The non-treated control animals received an equal dose of analgesia at the corresponding time point. Animal temperature was maintained with an infrared lamp controlled by feedback from a rectal temperature sensor, supplemented with a warm water pad. The tumor-bearing paw was positioned underneath the therapeutic transducer. The paw was fully covered with degassed ultrasound transmission gel (Aquasonic 100, Parker Laboratories, Fairfield, NJ, USA). An acoustic absorber (Aptflex F28P, Precision Acoustics, Dorchester, UK) was positioned underneath the paw to prevent far-field heating. A photograph and a schematic drawing of the HIFU set-up are shown in Figure S1.

Partial tumor ablation was performed such that both HIFU-treated and non-treated tumor tissue were present after treatment. Positioning of the tumor in the focal point of the therapeutic transducer was confirmed by use of an ultrasound imaging system (HDI5000 imaging system combined with a P7-4 phased array transducer, Philips Ultrasound, Bothell, WA, USA). Ultrasound imaging was solely used for treatment planning; ultrasound-based treatment monitoring was beyond the scope of the present study. A square 4×4 mm^2^ treatment grid consisting of 25 equally-spaced treatment points was defined within the tumor. A wait time of 120 s was applied between the point-wise HIFU treatments to allow sufficient cooling of the tissue. Treatment settings were: frequency = 1.4 MHz, pulse repetition frequency = 20 Hz, acoustic power = 12 W, duty cycle = 50%, treatment time = 30 s. In three pilot experiments, a thermocouple (T-type thermocouple; T-150A, Physitemp Instruments, Clifton, NJ, USA) was positioned in the focal point of the therapeutic transducer to monitor temperature during the treatment. The temperature increased to approximately 66°C during the sonication and decreased again to the pre-sonication temperature (approximately 35°C) during the wait time. A representative temperature profile is shown in Figure S2. The thermocouple was not inserted during the treatment of the experimental groups to prevent non-HIFU-related damage to the tumor tissue.

### MRI measurements

MRI measurements were performed with a 6.3 T scanner (Bruker BioSpin, Ettlingen, Germany) using a 3.2-cm-diameter quadrature birdcage RF coil (Rapid Biomedical, Rimpar, Germany). Anesthesia was maintained with 1–2% isoflurane during the MRI experiments. The mice were positioned in a custom-made cradle, equipped with a mask for anesthetic gas. The tumor-bearing paw was fixed in the set-up by adhesive tape in order to prevent motion artifacts. No motion was observed between the images of the different sequences and therefore no further post-processing image registration proved necessary. Respiration was monitored with a balloon sensor. Body temperature was monitored and maintained with a warm water pad. For reduction of susceptibility artifacts in the Echo Planar Imaging (EPI) sequence, the tumor-bearing paw was covered with degassed ultrasound gel (Aquasonic 100, Parker Laboratories). Artifacts were further reduced by local shimming of the tumor-bearing paw. To illustrate the obtained image quality of the EPI sequence, representative images of a conventional T_2_-weighted spin-echo acquisition and a T_2_-prepared gradient-echo EPI (GE-EPI) sequence are shown in Figure S3. No apparent geometric distortion artifacts were present in the EPI images.

The multi-slice MRI protocol, covering the whole tumor, started with a fat-suppressed T_2_-weighted spin-echo sequence (echo time TE = 30 ms, repetition time TR = 1000 ms, number of averages NA = 1) for anatomical reference. Subsequently, T_1_, T_2_, ADC and MTR mapping were performed. T_1_ mapping was performed with an inversion recovery Look-Locker EPI method (TE = 8 ms, TR = 10000 ms, inversion time = 30 ms, flip angle = 20°, pulse separation = 400 ms, number of points = 15, NA = 2). For T_2_ mapping, a T_2_-weighted MLEV-prepared [27] GE-EPI sequence (TR = 2000 ms, NA = 2) was acquired with 7 TE's ranging from 1 to 82 ms. For ADC mapping, a diffusion-weighted double spin-echo prepared EPI sequence (TE = 41 ms, TR = 4000 ms, NA = 4) was used to acquire images with 4 different b-values (0, 100, 200 and 400 s/mm^2^). The diffusion-sensitizing gradient was applied separately in three orthogonal directions. The MTR mapping protocol consisted of two GE-EPI acquisitions (TE = 8 ms, TR = 8000 ms, NA = 2) with and without an off-resonance preparation pulse (4000 ms block pulse, B_1_ = 1.3 µT, -10 ppm from water resonance frequency). All acquired images had a matrix size of 128×128, FOV of 4×4 cm^2^ and 1 mm slice thickness. Twelve to 16 slices were acquired covering the whole tumor volume.

### Image processing and generation of parameter maps

Image analysis was performed in Mathematica 7.0 (Wolfram Research, Champaign, IL, USA). Regions of interest (ROIs) were defined on the T_2_-weighted images by manually drawing contours around the tumor tissue on each slice. Diffusion-weighted images were used as an additional reference for tumor demarcation. Parameter maps were calculated on a pixel-by-pixel basis in each slice. T_1_ maps were generated as described previously [28]. T_2_ maps were calculated from mono-exponential fitting of the multi-echo data. For the generation of ADC maps, mono-exponential fitting was performed through the signal intensities at the different b-values for each diffusion-encoding direction separately. Next, ADC values of the different directions were averaged to obtain the final (orientation-invariant) ADC value for each pixel. MTR maps were generated according to MTR = (1-S/S_0_)*100%, in which S and S_0_ are the pixel signal intensities with and without off-resonance irradiation, respectively.

### ISODATA analysis

ISODATA cluster analysis was employed to segment the multiparametric data into groups of pixels, *i.e.* clusters, with similar MR parameter values. The ISODATA technique was implemented in Mathematica 7.0 according to the description given by Jacobs *et al*. [25]. A schematic overview of the ISODATA algorithm can be found in Supporting Information S1. The ISODATA clustering technique is similar to the widely applied clustering algorithm *k-*means [29]. However, as opposed to *k*-means, the number of clusters does not have to be determined *a priori* for the ISODATA algorithm. The number of clusters is rather adjusted iteratively according to the Euclidean distance between and within the clusters. ISODATA clustering was performed on the multi-slice parametric images of all animals (HIFU-treated and control) at all time points simultaneously. Prior to ISODATA clustering, features were normalized (mean µ = 0, standard deviation (SD) = 1) to remove scaling differences between the different parameters. Pixels of which the signal intensity in the T_2_-weighted images was at noise level, *e.g.* because of HIFU-induced hemorrhage, were excluded from ISODATA analysis. ISODATA clustering was performed on the following subsets of MRI parameters, termed feature vectors: {T_2_}, {ADC}, {T_1_,T_2_}, {T_2_,ADC}, {T_1_,ADC}, {ADC,MTR}, {T_1_,T_2_,ADC}, {T_2_,ADC,MTR}, {T_1_,ADC,MTR}, {T_1_,T_2_,MTR} and {T_1_,T_2_,ADC,MTR}. The resulting clusters were divided into two different classes:

- Non-viable: clusters of which the fraction of assigned pixels increased significantly after HIFU (either at 1 h or 72 h) compared to before HIFU (paired Student's t-test, p<0.05);
- Viable: all remaining clusters.

Subsequently, all tumor pixels of both the HIFU-treated and non-treated animals at all experimental time points were assigned as either viable or non-viable based on the class of the cluster to which the pixel belongs.

**Figure 1:**
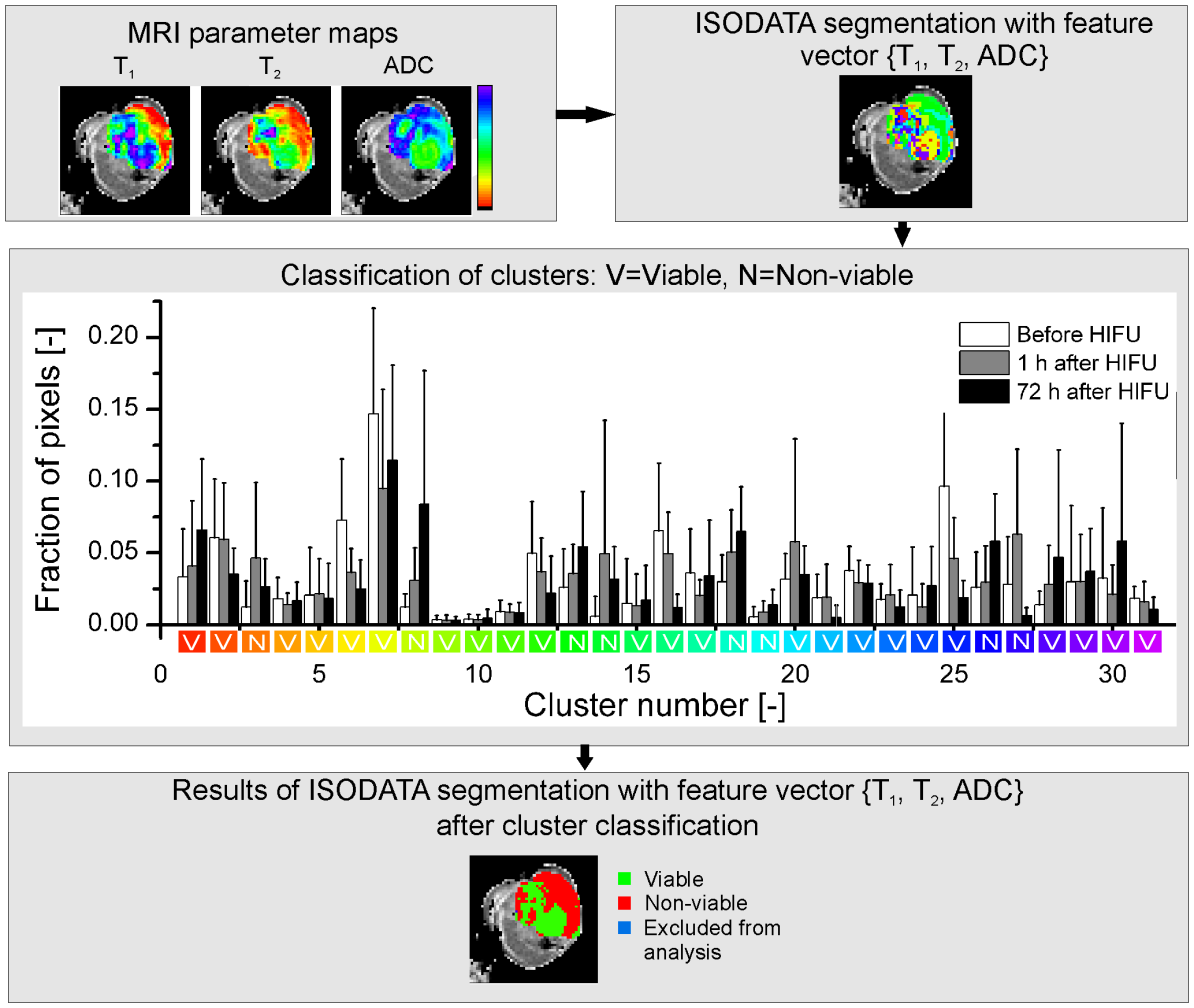
Flow chart of ISODATA segmentation. Flow chart of the classification of clusters resulting from ISODATA segmentation with feature vector {T_1_, T_2_, ADC}. MRI parameter maps were generated from the multiparametric MRI data (top left). The displayed MRI results originate from a tumor scanned at 72 h after HIFU treatment. Pixels were clustered into tissue populations with similar MRI parameters (top right; different colors represent different clusters). The resulting clusters were classified as either viable (labeled ‘V’ in the histogram) or non-viable (labeled ‘N’ in the histogram) based on the fraction of pixels assigned to the clusters at the different time points. Clusters of which the fraction of pixels had increased significantly (paired Student's t-test, p<0.05) after HIFU compared to before HIFU were assigned to non-viable tumor tissue. The remaining clusters were assigned to viable tumor tissue. The histogram of the fractions of tumor pixels in each cluster at the different time points is displayed in the center of the figure. The white, grey and black bars represent mean ± SD of the fractions of tumor pixels before, at 1 h after and at 72 h after HIFU, respectively. The color coding on the x-axis of the histogram corresponds to the cluster colors in the ISODATA segmentation results (top right). The result of the ISODATA segmentation after classification of the clusters as either viable or non-viable tumor tissue is shown in the bottom part of the figure. A small number of tumor pixels was excluded from ISODATA analysis, because of a low signal-to-noise ratio (see Methods).

A schematic view of the classification of the clusters into either non-viable or viable tumor tissue is given in Figure 1.

Based on this classification, ISODATA-derived non-viable tumor volume fractions were calculated for each tumor for the different feature vectors. These tumor fractions were compared to histology-derived non-viable tumor volume fractions in order to select the optimal feature vector, *i.e*. the combination of MRI indices, which led to the best agreement with the histological differentiation between non-viable and viable tumor tissue.

Mean MRI parameter values in the pixels classified as viable and non-viable tumor tissue were calculated to quantify the effects of HIFU treatment on the measured MRI parameters.

### Histological analysis

Dissected tumors were snap-frozen in isopentane and stored at −80°C. Tumors were cut into 5 µm thick sections with a distance of approximately 300 µm between the sections. The cryo-sections were briefly air-dried and subsequently stained for nicotinamide adenine dinucleotide (NADH) diaphorase activity to assess cell viability. NADH-diaphorase staining is a powerful histological tool for demarcation between viable and non-viable tumor tissue after HIFU treatment [30]. Sections were incubated at 37°C for 1 h in Gomori-Tris-HCl buffer (pH 7.4) containing β-NAD reduced disodium salt hydrate (Sigma-Aldrich, St. Louis, MO, USA, 0.71 mg/ml buffer solution) and nitro blue tetrazolium (Sigma-Aldrich, 0.29 mg/ml buffer solution). Brightfield microscopy was performed on all sections and consisted of mosaic acquisition of the entire section at 5× magnification.

Analysis of the microscopy images was performed in Mathematica 7.0. ROIs were manually drawn around the pale non-viable tumor tissue and the entire tumor tissue on all sections of each tumor. From the ratio between the ROI areas of non-viable tumor tissue and entire tumor tissue on all tumor sections, a histology-derived non-viable tumor volume fraction was determined for each tumor.

### Statistical analysis

Data are reported as mean±SD. For the determination of the optimal feature vector, non-viable tumor fractions derived from ISODATA segmentation with the different feature vectors were quantitatively compared to the histology-derived non-viable tumor volume fractions. Initial feature vector selection was based on the one-to-one correspondence between histology-derived and ISODATA-derived non-viable tumor fractions. The one-to-one correspondence was determined by calculation of the coefficient of determination (R^2^) of the data points, consisting of the ISODATA-derived and the histology-derived non-viable tumor fraction of each tumor, to the line of identity (y = x). This initial selection led to the elimination of feature vectors for which the ISODATA-derived non-viable tumor fractions were either strongly over- or underestimated compared to the histology-derived non-viable tumor fractions. Subsequently, Pearson's correlation coefficients were determined between the histology-derived and ISODATA-derived tumor fractions, as a measure for the strength of the linear relationship between the histology-derived and ISODATA-derived non-viable tumor fractions. These correlation values were determined for two different groups of animals: one group consisting of the animals sacrificed after the MRI examination at 1 h after HIFU and the non-treated control animals (referred to as ‘1 h after HIFU + Control’) and one group consisting of the animals sacrificed after the MRI examination at 72 h after HIFU and the control animals (referred to as ‘72 h after HIFU + Control’). This division in groups was made to take into account the temporal changes in tumor tissue after HIFU treatment, which might lead to a different optimal feature vector for the different time points after HIFU. The control animals were included in both groups to increase the statistical power and to obtain a larger range of non-viable tumor fractions. Differences in correlation values between the different feature vectors were tested for significance with a Wolfe's test for Comparing Dependent Correlation Coefficients [31].

For the HIFU-treated animals, the MRI parameter values in ISODATA-defined non-treated tumor tissue at all time points were compared to the parameter values in ISODATA-defined non-viable tumor tissue at 1 h and 72 h after HIFU with a paired Student's t-test. For all tests the level of significance was set at α = 0.05.

## Results

### MRI parameter maps

**Figure 2:**
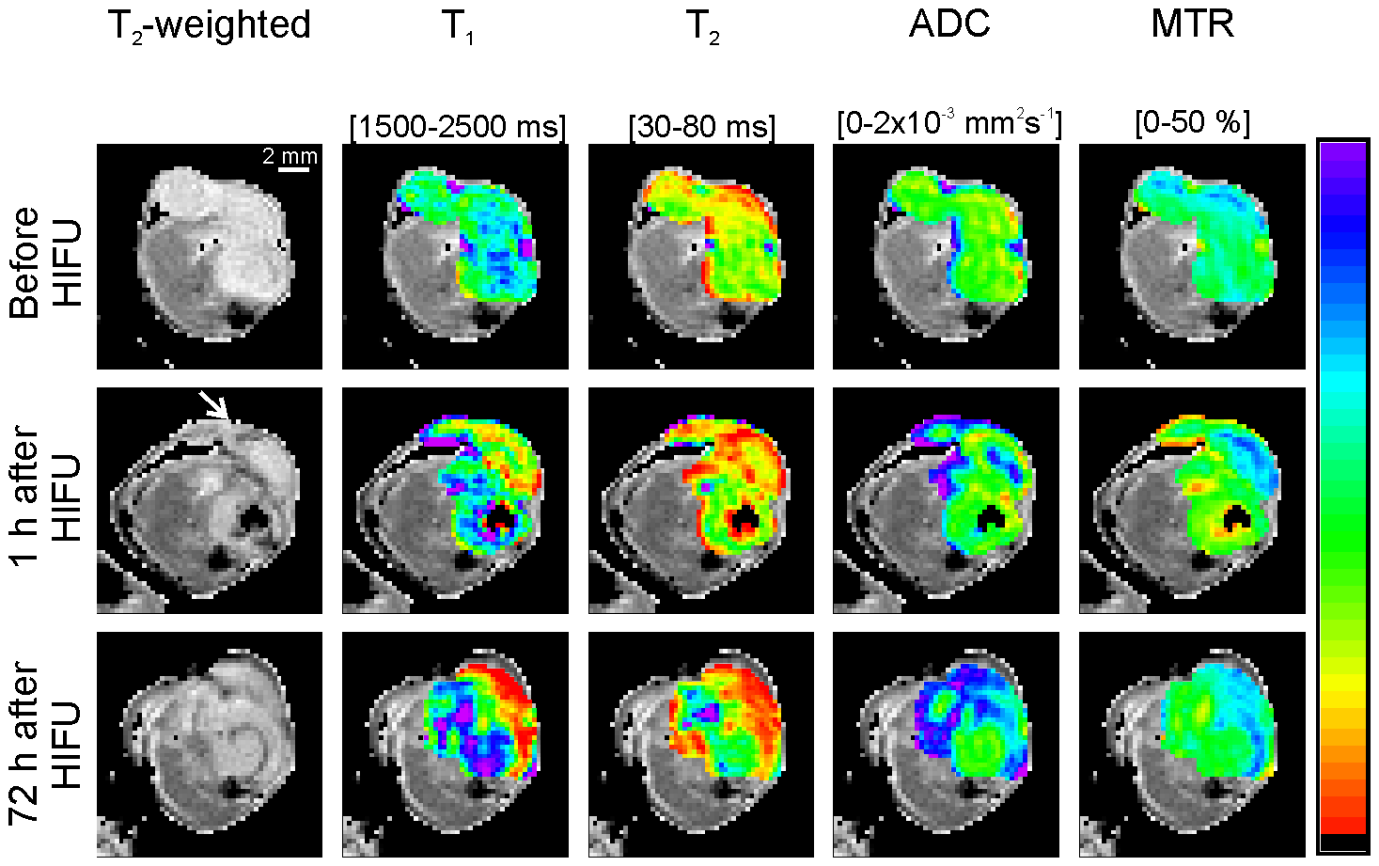
MRI parameter maps before and longitudinally after HIFU treatment. Representative example of multiparametric MRI of the hind limb region of a HIFU-treated tumor-bearing mouse before and 1 h and 72 h after HIFU treatment. T_2_-weighted images of an axial slice of the tumor-bearing paw are shown in the left panel. The hyper-intense tumor tissue is surrounded by hypo-intense muscle tissue. In the other panels the same T_2_-weighted images are displayed except that the tumor pixels are overlaid with MRI parameter maps. The parameter maps were scaled according to the color scale bar shown at the right-hand side of the figure. The corresponding parameter range for this scale bar is indicated above each panel. The approximate direction of the HIFU treatment is shown by the white arrow on the T_2_-weighted image, which was collected 1 h after HIFU treatment.

Representative MRI parameter maps as measured before, 1 h and 72 h after HIFU treatment are shown in Figure 2. A heterogeneous appearance of the HIFU-treated lesion was visible on the T_2_-weighted images at both time points after HIFU treatment. Distinct regions with decreased T_1_ and T_2_ were observed at 1 h after HIFU. A further increase in the area of T_1_ and T_2_ decline was observed in these regions at 72 h after HIFU. Increased ADC and MTR values were observed in roughly the same regions of T_1_ and T_2_ change, both at 1 h and 72 h after HIFU. However, no sharp demarcation between HIFU-treated and non-treated tumor tissue was visible on the individual MRI parameter maps obtained after HIFU. Tumor regions with altered MRI parameters co-localized only partially for the different parameters. This implied that more advanced analysis of the multiparametric data is necessary to enable MRI-based identification of the HIFU-treated tumor tissue. Therefore, quantitative multiparametric analysis of the MRI parameter changes after HIFU treatment was performed using the ISODATA technique. Clusters resulting from the ISODATA segmentation were classified as either viable or non-viable based on the fraction of pixels assigned to the clusters at the different experimental time points (Figure 1). ISODATA clustering was applied on different combinations of the MRI parameters (*i.e.* feature vectors), to determine the feature vector that led to the most accurate segmentation between viable and non-viable tumor tissue.

### Feature vector selection

Selection of the optimal feature vector for the discrimination between HIFU-treated, non-viable and non-treated, viable tumor tissue was performed based on quantitative comparison between non-viable tumor fractions resulting from ISODATA segmentation with different feature vectors and non-viable tumor fractions derived from whole-tumor-based histology.

**Figure 3:**
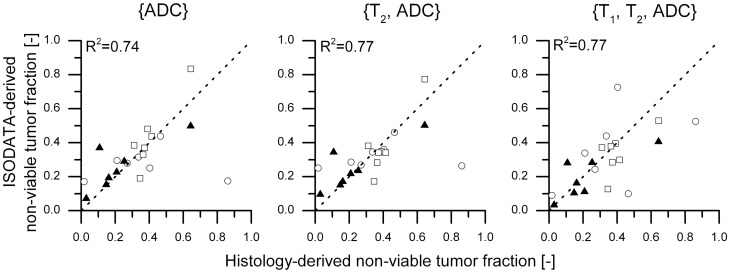
One-to-one correspondence between histology-derived and ISODATA-derived non-viable tumor fractions. Scatter plots of the ISODATA-derived non-viable tumor fractions following segmentation with feature vectors {ADC}, {T_2_, ADC} and {T_1_, T_2_, ADC} as a function of the histology-derived non-viable tumor fractions. The symbols ○, □ and ▴ indicate groups ‘1 h after HIFU’, ‘72 h after HIFU’ and ‘Control’, respectively. The line of identity is shown as visual reference. The R^2^ values of the data to the line of identity are shown in the top left corner of each plot.

Plots of the histology-derived non-viable tumor fractions versus the ISODATA-derived non-viable tumor fractions were processed for all assessed feature vectors. The R^2^ of the data points to the line of identity, indicative of the level of one-to-one correspondence between the results from ISODATA analysis and histology, was used as an initial criterion for feature vector selection. R^2^ values for all assessed feature vectors are listed in Table 1. Relatively high R^2^ values (>0.7) were observed for three feature vectors: {ADC}, {T_2_, ADC} and {T_1_, T_2_, ADC}, which were therefore considered candidates for the segmentation of HIFU-treated tumor tissue. Scatter plots of the ISODATA-derived and histology-derived non-viable tumor fractions are displayed in Figure 3 for these candidate feature vectors.

**Table 1:** R^2^ values of ISODATA-derived versus histology-derived non-viable tumor fractions to the line of identity for all assessed feature vectors.

Feature vector	R^2^ to line of identity [-]
{T_2_}	0.60
{ADC}	0.74
{T_1_, T_2_}	0.70
{T_2_, ADC}	0.77
{T_1_, ADC}	0.68
{ADC, MTR}	−1.67
{T_1_, T_2_, ADC}	0.77
{T_2_, ADC, MTR}	−0.09
{T_1_, ADC, MTR}	0.42
{T_1_, T_2_, MTR}	−0.79
{T_1_, T_2_, ADC, MTR}	−0.16

**Figure 4:**
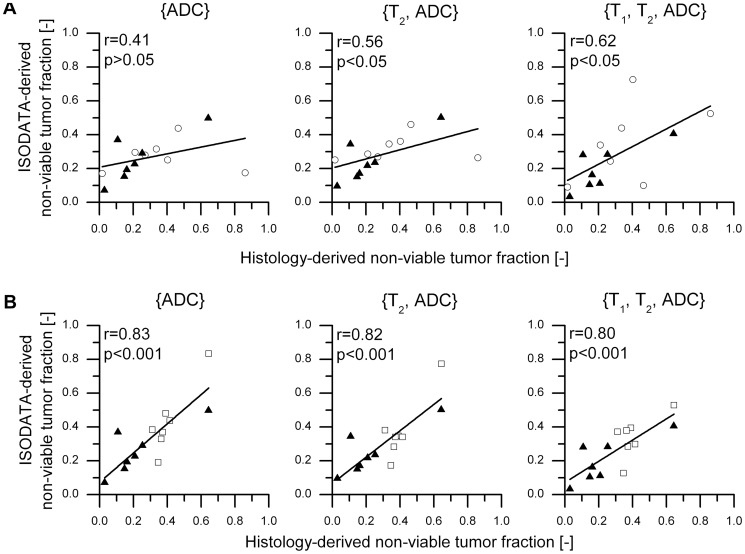
Correlation between histology-derived and ISODATA-derived non-viable tumor fractions. Correlation plots of ISODATA-derived non-viable tumor fractions following segmentation with feature vectors {ADC}, {T_2_, ADC} and {T_1_, T_2_, ADC} as a function of the histology-derived non-viable tumor fractions for two different groups of animals: ‘1 h after HIFU + Control’ (**A**) and ‘72 h after HIFU + Control’ (**B**). The symbols ○, □ and ▴indicate groups ‘1 h after HIFU’, ‘72 h after HIFU’ and ‘Control’, respectively. Correlation values between the ISODATA-derived and the histology-derived tumor fractions are listed in the top left corner of each plot.

The correlation between the histology-derived and ISODATA-derived non-viable tumor fractions was used as a second criterion for feature vector selection. The experimental groups were divided into two groups for this correlation analysis: ‘1 h after HIFU + Control’ and ‘72 h after HIFU + Control’ (see Methods). Correlation plots for the three candidate feature vectors are depicted in Figure 4 for both groups. For group ‘1 h after HIFU + Control’ the strongest correlation was found for feature vector {T_1_, T_2_, ADC} (r = 0.62, moderate correlation; Figure 4A). However, for this group, no statistically significant differences between the correlation values of the different candidate feature vectors were observed. A strong correlation was observed for all three feature vectors for group ‘72 h after HIFU + Control’ (Figure 4B). The strongest correlation in this case was found for feature vector {ADC} (r = 0.83), which was significantly higher than the correlation value found for feature vector {T_1_, T_2_, ADC} (r = 0.80).

The third criterion for feature selection consisted of minimization of the number of pixels incorrectly assigned to non-viable tissue before ablation. Most notably for the feature vectors {ADC} and {T_2_, ADC}, a portion of pixels in the tumor rim was incorrectly assigned to non-viable tissue before HIFU, whereas this was observed to a lesser extent for feature vector {T_1_, T_2_, ADC}. The incorrect classification of the tumor pixels was caused by presence of peritumoral edema at the tumor rim. Visual inspection during excision of the tumors confirmed presence of edema around the HIFU-treated tumors. The fraction of rim pixels (rim thickness of 3 pixels) that was incorrectly assigned as non-viable before HIFU application was therefore used as a further selection measure. The fractions of incorrectly assigned rim pixels were significantly lower for feature vector {T_1_, T_2_, ADC} (0.15±0.09) as compared to feature vectors {ADC} and {T_2_, ADC} (0.28±0.10 and 0.25±0.08, respectively).

Based on the above three criteria, feature vector {T_1_, T_2_, ADC} provided the optimal combination of MRI parameters for differentiation between HIFU-treated and non-treated tumor tissue.

### Evaluation of MRI parameter changes

**Figure 5:**
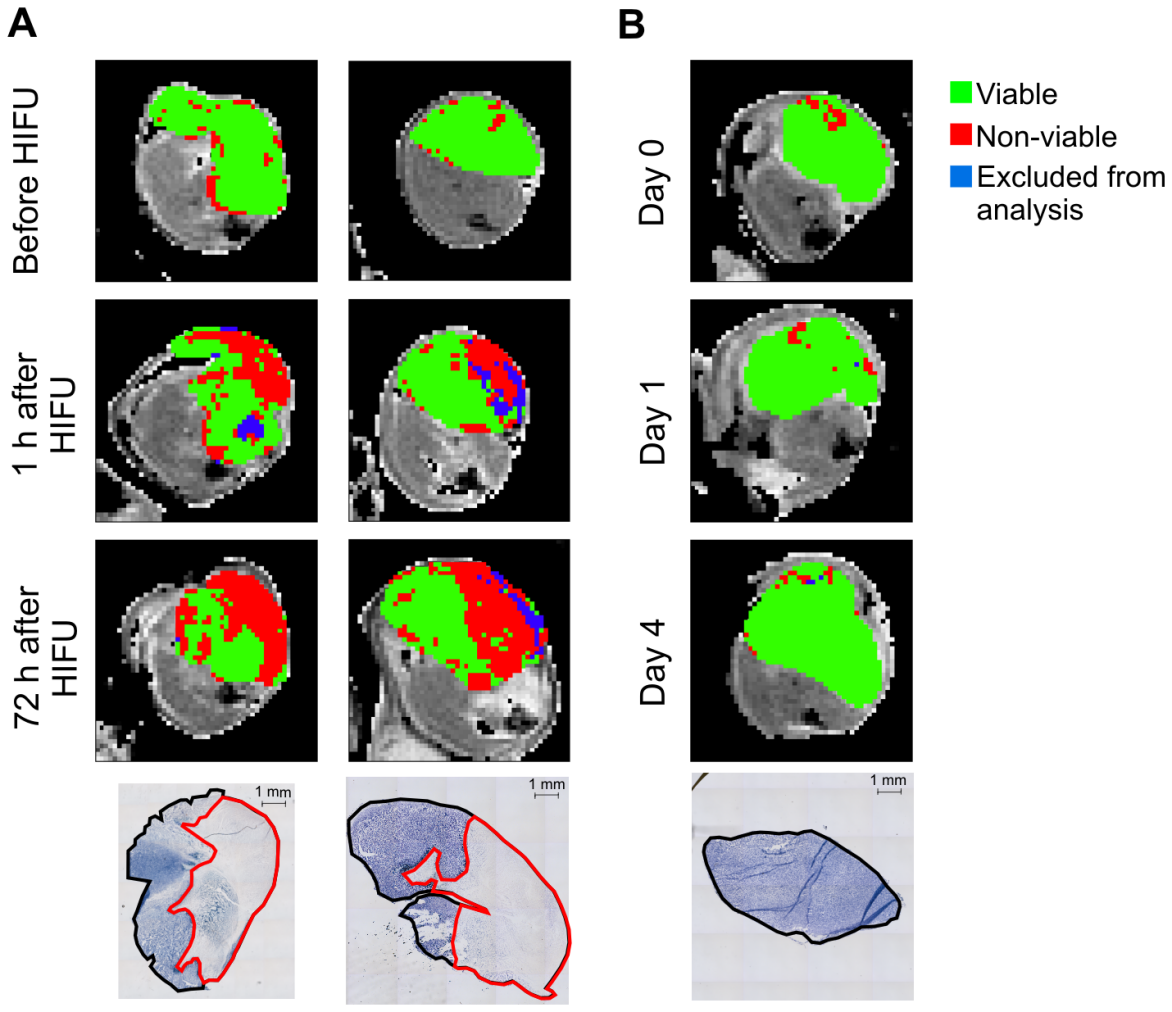
Visual correspondence between ISODATA-derived and histology-derived non-viable tumor areas. **A**) Representative T_2_-weighted images of the hind limb region of two HIFU-treated mice before, 1 h after and 72 h after HIFU. The results of ISODATA segmentation with feature vector {T_1_, T_2_, ADC} are overlaid on the tumor pixels. NADH-diaphorase stained sections of tumors dissected at 72 h after HIFU were made at approximately the same location within the tumor and are shown at the bottom of each column. ROIs around the entire (black line) and non-viable (red line) tumor tissue were drawn manually. Data in the left column are from the animal presented in Figure 1. **B**) Similar data of a non-treated control mouse that was subjected to serial MRI measurements at the same time points (Day 0, Day 1 and Day 4) as the HIFU-treated animals. Scale bar = 1 mm.

In Figure 5A representative results of the ISODATA segmentation with feature vector {T_1_, T_2_, ADC} are displayed for two HIFU-treated animals. As anticipated, at baseline the largest fraction of pixels (0.85±0.10 for all HIFU-treated animals) was assigned to viable tumor tissue. At 1 h after HIFU a region emerged in which pixels were assigned to HIFU-treated, *i.e.* non-viable tumor tissue. At 72 h after HIFU this region had grown and showed good spatial agreement with a region of non-viable tumor tissue on an NADH-diaphorase stained section at approximately the same location within the tumor. A 3D reconstruction of the ISODATA segmentation of the tumor displayed in the right panel of Figure 5A at 72 h after HIFU can be seen in the supporting content (Movie S1). This 3D reconstruction was processed by interpolation of the tumor pixel data resulting from the ISODATA segmentation. The reconstruction shows a distinct, contiguous non-viable, HIFU-treated tumor volume surrounded by viable tumor tissue. In comparison, Figure 5B shows representative results of ISODATA segmentation of a non-treated control animal that was subjected to MRI examination at the same time points. Here only a small number of pixels was assigned to non-viable tumor tissue at all time points, in agreement with a fully viable tumor observed by NADH-diaphorase staining.

Average MRI parameter values of ISODATA-defined viable tumor tissue at all experimental time points and non-viable tumor tissue at both time points after HIFU are listed in Table 2 for feature vector {T_1_, T_2_, ADC}. R_1_ (1/T_1_), R_2_ (1/T_2_), ADC and MTR were significantly increased in the non-viable, HIFU-treated tumor tissue 1 h after HIFU compared to viable tumor tissue. R_1_, ADC and MTR remained significantly increased in non-viable tumor tissue 72 h after HIFU. No significant differences in parameter values were observed between 1 h and 72 h after HIFU treatment.

**Table 2:** Mean MRI parameter values in ISODATA-defined viable and non-viable tumor tissue.

MRI parameter	Viable tumor tissue	Non-viable tumor tissue 1 h after HIFU	Non-viable tumor tissue 72 h after HIFU
**R_1_ [s^−1^]**	0.45±0.01	0.61±0.13 **	0.60±0.06 **
**R_2_ [s^−1^]**	21.7±1.4	35.8±19.5 **	34.0±21.0
**ADC [10^−3^ mm^2^s^−1^]**	0.84±0.12	1.09±0.20 **	1.25±0.16 **
**MTR [%]**	23.3±1.2	26.4±2.7 **	26.4±3.5 *

MRI parameter values (mean±SD) in viable tumor tissue (tumor tissue assigned as viable tumor tissue at all time points (n = 14)), non-viable tumor tissue at 1 h after HIFU (n = 14) and non-viable tumor tissue at 72 h after HIFU (n = 7) of the HIFU-treated animals following ISODATA segmentation with feature vector {T_1_, T_2_, ADC}. * and ** denote a significant difference between viable and non-viable tumor tissue with p<0.05 and p<0.001, respectively (paired Student's t-test).

## Discussion and Conclusions

In the present study multiparametric MR analysis was performed to distinguish non-viable, HIFU-treated tumor tissue from viable, non-treated tumor tissue in a murine tumor model. The longitudinal multiparametric MRI measurements consisted of quantitative assessments of T_1_, T_2_, ADC and MTR. ISODATA segmentation was applied on various feature vectors. ISODATA-derived non-viable tumor volume fractions were compared to non-viable tumor fractions derived from quantitative histology to identify the optimal feature vector for differentiation between non-viable, HIFU-treated and viable, non-treated tumor tissue.

The most accurate distinction between HIFU-treated and non-treated tumor tissue was obtained with feature vector {T_1_, T_2_, ADC}. For this feature vector, the correlation between the histology-derived and ISODATA-derived fractions of non-viable tumor tissue was lower for group ‘1 h after HIFU + Control’ than for group ‘72 h after HIFU + Control’. These results suggest that time is needed before HIFU-induced changes in the tumor tissue produce sufficient detectable contrast in the MRI images.

A multiparametric protocol consisting of T_1_, T_2_ and ADC mapping was sufficient to segment HIFU-treated and non-treated tumor tissue. Inclusion of MTR to the protocol led to less agreement of the ISODATA segmentation with the histological analysis. Nevertheless, a significant increase in MTR was observed in the non-viable tumor tissue identified from ISODATA analysis with feature vector {T_1_, T_2_, ADC} (Table 2). However, since this increase was only subtle, clustering with inclusion of MTR led to merging of clusters of non-viable and viable tumor tissue, resulting in a lower specificity and sensitivity of the cluster method.

ISODATA clustering with feature vector {T_1_, T_2_, ADC} yielded 31 different clusters (Figure 1), which indicated a large heterogeneity of the HIFU-treated tumor tissue. Extensive analysis of each cluster separately would yield additional information about the status of the different segmented tissue populations. However, the focus of this study was the binary distinction between non-viable, HIFU-treated and viable, non-treated tumor tissue. Therefore, it was decided to allocate the clusters resulting from ISODATA segmentation to two distinct classes, designated as non-viable and viable. A cluster was assigned to the non-viable class if the fraction of pixels within the cluster significantly increased by HIFU treatment. This criterion could however also be met if the cluster is associated with reversible HIFU-induced tissue changes instead of with non-viable tumor tissue. Nevertheless, since a high one-to-one correspondence between histology-derived and ISODATA-derived non-viable tumor fractions was observed for the optimal feature vector {T_1_, T_2_, ADC}, it seems reasonable to assume that the majority of the non-viable clusters for that feature vector indeed represents non-viable tumor tissue.

A minor part of the tumor pixels after HIFU treatment (fractions of 0.05±0.03 and 0.02±0.02 of the entire tumor tissue at 1 h and 72 h after HIFU, respectively) needed to be excluded from the multiparametric MR analysis, because the signal intensity of these tumor pixels was at noise level in the T_2_-weighted images (see Methods). This low signal intensity was most likely caused by HIFU-induced hemorrhage. Exclusion of these hemorrhage-associated pixels from the analysis did not influence the correlation results presented here, because it only involved a very low fraction of tumor pixels.

A reconstruction of the ISODATA segmentation results was made to visualize the HIFU-treated volume in 3D space (Movie S1). A similar reconstruction of the histological data and registration of these data with the MRI findings would allow for analysis of the spatial correlation between the ISODATA-segmented and histology-based non-viable volumes. However, 3D reconstruction of histological tumor sections was not performed in the present study. Accurate 3D histological reconstruction and its registration with MRI would have required an intermediate MRI scan after tumor excision, a robust anatomical reference and denser histological sampling [32]. Additionally, spatial correlation of histology with MRI would require a higher spatial resolution of the MR images. This would lead to a substantially longer acquisition time of the multiparametric MR protocol and result in unacceptably long anesthesia times for the animals.

Instead of assessment of spatial correlation, the ISODATA analysis method was optimized based on the correlation between histology-derived and ISODATA-derived non-viable tumor fractions for different feature vectors. A similar correlation analysis has been performed for the detection of necrotic tumor tissue after radiotherapy, which yielded comparably strong correlations between clustering and histology [33], showing that this global correlation analysis is suitable for the optimization of clustering methods. For the optimized feature vector {T_1_, T_2_, ADC}, a strong spatial agreement between the region of ISODATA-derived non-viable tumor tissue and the region of non-viable tumor tissue on NADH-diaphorase-stained histological sections (Figure 5) was observed visually, which indicates that the proposed methodology allows for accurate segmentation of HIFU-treated, non-viable tumor tissue.

Mean MRI parameter values were determined in non-viable and viable tumor tissue following ISODATA segmentation with feature vector {T_1_, T_2_, ADC} (Table 2). R_1_ was significantly increased in the non-viable, HIFU-treated tumor tissue both at 1 h and 72 h after HIFU as compared to viable, non-treated tumor tissue. Such increased R_1_ (or decreased T_1_) was previously also observed *ex vivo* in (non-cancerous) tissues after heat treatment [34], which was explained by a combination of coagulation of the blood volume fraction and disruption of biological barriers, which facilitates access of water molecules to paramagnetic sites in the coagulated blood. Decreased T_1_ was also observed as a result of tissue necrosis after chemotherapy [35].

Graham *et al.* observed an increase in T_2_ after heating of *ex vivo* tissue, which was explained as vacuolization of water molecules caused by increased hydrophobic interactions induced by protein denaturation [34]. We observed significantly increased R_2_ (and thus decreased T_2_) in non-viable tumor tissue 1 h after HIFU treatment, whereas at 72 h after HIFU treatment no significant R_2_ differences between non-viable and viable tumor tissue were observed. This initially decreased T_2_ could be explained by the aforementioned increased access of water molecules to paramagnetic centers in the coagulated blood. Furthermore, the large standard deviation of R_2_ in the non-viable, HIFU-treated tumor tissue (Table 2) indicated large T_2_ heterogeneity due to the presence of areas with increased and decreased T_2_.

The observed increased ADC values in the non-viable tumor tissue have been described earlier *ex vivo* in thermally treated tissue samples [34] and *in vivo* in preclinical experiments on the effects of HIFU treatment in a murine tumor model [36] and in clinical studies of RF ablation of liver lesions [19]. Diffusion of water molecules could have been facilitated by HIFU-induced disruption of obstructing barriers, leading to an increased ADC.

Reports on the effects of thermal treatment on the MTR are conflicting. Both a decreased [34] and increased [23] MTR have been observed in *ex vivo* thermally treated samples. An MTR decrease could be explained by denaturation of structural proteins [34], whereas an increase might be caused by increased access of water molecules to macromolecules due to disruption of barriers. Furthermore, T_1_ effects may play a role, since the MTR is known to be affected by tissue T_1_ [37]. In the current study, a combination of these factors has probably influenced the observed MTR, which is also illustrated by the subtle yet significant increase in MTR in the non-viable, HIFU-treated tumor tissue compared to viable tumor tissue.

The HIFU treatment was performed outside the MR system, since the preclinical therapeutic ultrasound transducer is not (yet) MRI-compatible. In an MRI-guided HIFU set-up, results from the multiparametric MR analysis could be quantitatively compared to thermal dose maps derived from MR thermometry during HIFU treatment. Furthermore, the MRI examinations before and directly after HIFU could then be combined within the same anesthesia period. This was not feasible in this study, since the ablation procedure was rather lengthy for the current set-up (∼1 hour) and the subject would have to be positioned in the MR system twice. Nevertheless, no significant differences in MRI parameters were observed between the measurements of the control animals at Day 0 and Day 1, corresponding in time with the measurements of the HIFU-treated animals before and at 1 h after HIFU. This indicated that the time between the examinations before and after HIFU did not influence the results presented here.

A murine tumor model was used to assess the effects of HIFU treatment on tumor tissue status. The CT26 colon carcinoma was chosen because previous studies have shown that subcutaneous inoculations of CT26 cell suspensions lead to well-vascularized tumors [38] with limited necrosis [39]. This is beneficial for the current study, because extensive natural necrosis could mask the effects of HIFU-induced necrosis. However, the method is not restricted to this tumor model, since similar approaches, consisting of multispectral MR imaging combined with cluster analysis, have been employed for the identification of viable and non-viable tissue after chemotherapy [40] and radiotherapy [33] in other preclinical tumor models.

Clinical translation of the multiparametric protocol is feasible, since MR sequences for acquisition of the proposed MRI parameters, T_1_, T_2_ and ADC, are already clinically available. The total acquisition time of the multi-slice T_1_, T_2_ and ADC protocol was approximately 25 minutes. Further reduction of the measurement time would facilitate inclusion of the multiparametric MR measurements in clinical HIFU treatment protocols. The MR parameter mapping acquisitions could be accelerated by for example rapid imaging techniques, such as parallel imaging and compressed sensing. Prior to clinical introduction of the proposed multiparametric MR analysis, it would be necessary to further confirm that the ISODATA-derived non-viable tumor tissue spatially corresponds to non-viable tumor tissue in histology. This could for example be achieved by image-guided biopsies from the ISODATA-derived viable and non-viable tumor tissue. Since clinical trials on HIFU treatment of malignant tumors generally use treat-and-resect protocols [41], it would be feasible to include image-guided biopsies in the workflow of these studies. Such extensive validation should be performed in different tumor subtypes to assess the full clinical potential of the proposed methodology.

In summary, we have shown that non-viable, HIFU-treated tumor tissue could be distinguished from non-treated tumor tissue using quantitative multiparametric MRI combined with the ISODATA clustering technique. Clustering with feature vector {T_1_, T_2_, ADC} yielded a strong correlation between ISODATA-derived and histology-derived non-viable tumor fractions. The presented methodology not only offers clear insights in the HIFU-induced changes in MRI parameters, but could also ultimately be made suitable for clinical application and might offer the unique possibility to automatically detect residual or recurring tumor tissue after HIFU treatment in an objective manner. Furthermore, since the proposed MRI analysis is solely based on endogenous contrast, immediate retreatment is possible when residual or recurring tumor tissue is detected, without the risk of inaccurate temperature mapping due to presence of a Gd-based contrast agent.

## Supporting Information

Figure S1 **HIFU set-up.** *Left* Photograph of the HIFU set-up. The animal was positioned underneath the acoustic coupler. Animal temperature was maintained with an infrared lamp with temperature feedback control from a rectal temperature probe. The motion control stage allowed for accurate movement of the therapy transducer between the pre-defined treatment points. *Right* Schematic drawing of the HIFU set-up, showing positioning of the tumor tissue in the focus point of the therapy transducer. The surrounding hind limb tissue was positioned outside the focal zone.
(TIF)

Figure S2 **Temperature profile.** Typical example of a temperature profile during HIFU treatment. The tumor tissue temperature increased to 66°C during the sonication of 30 seconds, followed by cooling of the tissue to pre-sonication temperature during the wait time of 120 seconds. The temperature information was acquired by a thermocouple, which was inserted into the tumor tissue during the pilot experiments. The focal point of the therapeutic transducer co-localized with the tip of the thermocouple. This co-localization was verified by multiple low-power sonications around the expected thermocouple position. The exact thermocouple position was determined as the position at which the highest temperature increase was observed during the sonication.
(TIF)

Figure S3 **EPI image quality.** Two representative examples of conventional T_2_-weighted spin-echo images (left column of panels) and T_2_-prepared GE-EPI images (right column of panels). The effective echo times are similar for both images (30 ms for the conventional T_2_-weighted images; 28 ms for the T_2_-prepared GE-EPI images). Regions of interest (ROIs) of the tumor tissue are indicated with the red lines, showing absence of apparent geometric distortion within the tumor tissue in the EPI images.
(TIF)

Movie S1 **3D reconstruction of results from ISODATA segmentation.**
(AVI)

Supporting Information S1 **Theory: the ISODATA clustering algorithm.**
(PDF)
