# Supplementary figures and images for: Multiparametric MRI Analysis for the Identification of High Intensity Focused Ultrasound-Treated Tumor Tissue
Source: PLoS One. 2014 Jun 13;9(6):e99936. doi: 10.1371/journal.pone.0099936 (PMC4057317; doi:10.1371/journal.pone.0099936)

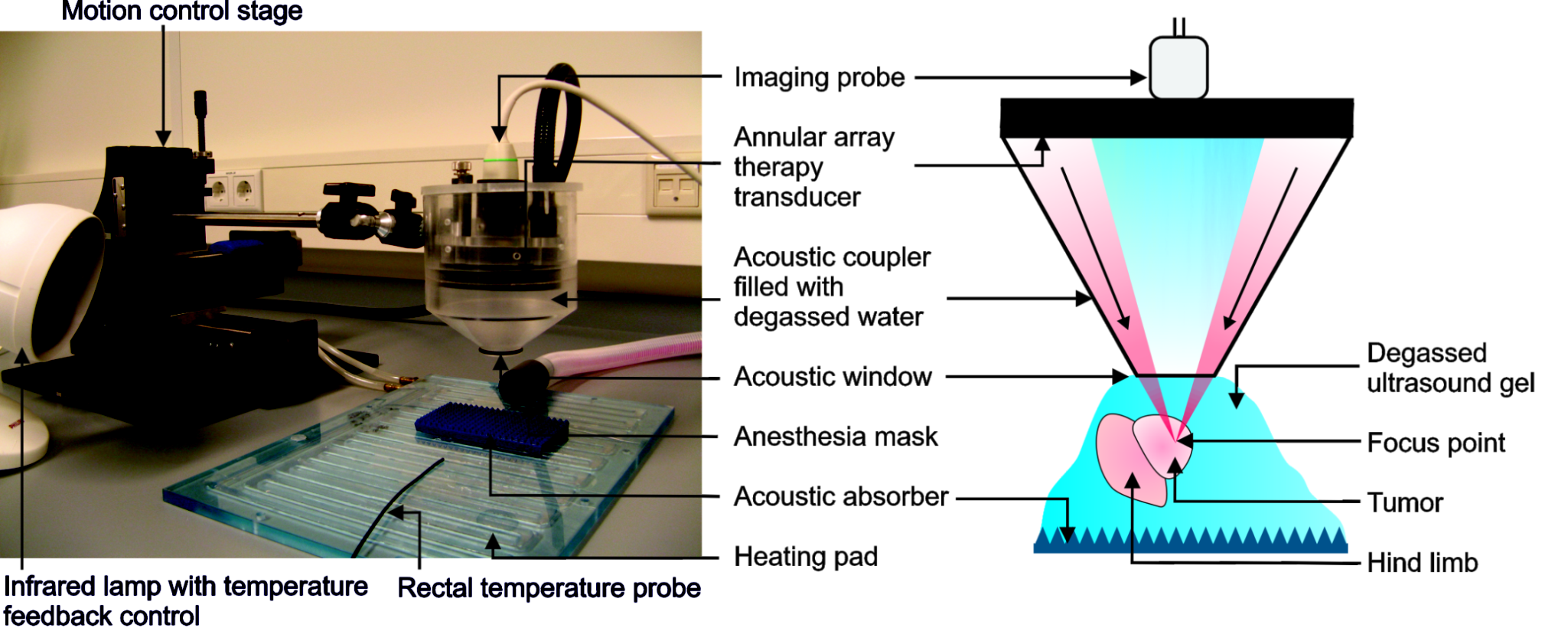

Supplement: Figure S1 — HIFU set-up. Left Photograph of the HIFU set-up. The animal was positioned underneath the acoustic coupler. Animal temperature was maintained with an infrared lamp with temperature feedback control from a rectal temperature probe. The motion control stage allowed for accurate movement of the therapy transducer between the pre-defined treatment points. Right Schematic drawing of the HIFU set-up, showing positioning of the tumor tissue in the focus point of the therapy transducer. The surrounding hind limb tissue was positioned outside the focal zone. (TIF) [file pone.0099936.s001.tif]

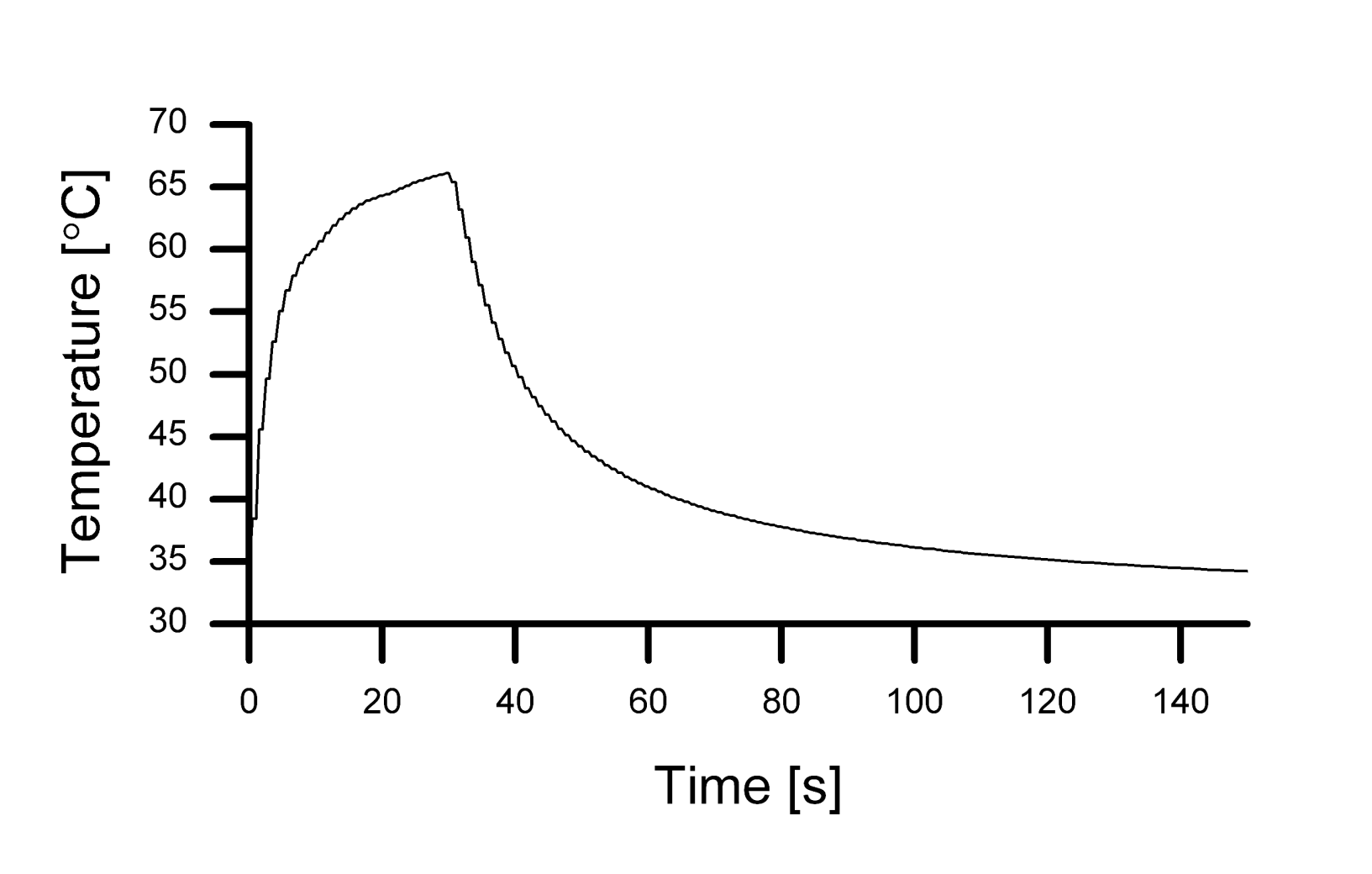

Supplement: Figure S2 — Temperature profile. Typical example of a temperature profile during HIFU treatment. The tumor tissue temperature increased to 66°C during the sonication of 30 seconds, followed by cooling of the tissue to pre-sonication temperature during the wait time of 120 seconds. The temperature information was acquired by a thermocouple, which was inserted into the tumor tissue during the pilot experiments. The focal point of the therapeutic transducer co-localized with the tip of the thermocouple. This co-localization was verified by multiple low-power sonications around the expected thermocouple position. The exact thermocouple position was determined as the position at which the highest temperature increase was observed during the sonication. (TIF) [file pone.0099936.s002.tif]

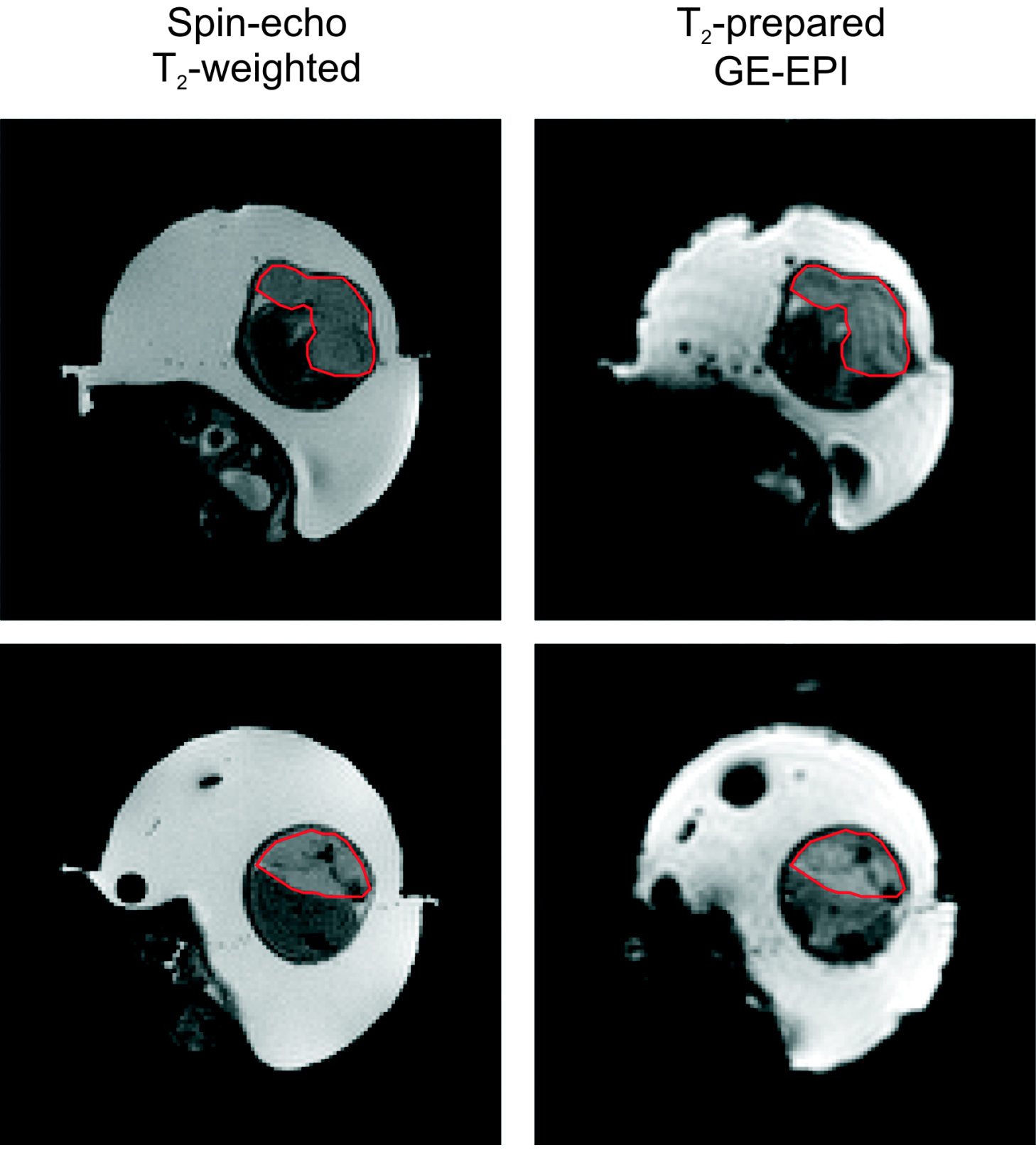

Supplement: Figure S3 — EPI image quality. Two representative examples of conventional T2-weighted spin-echo images (left column of panels) and T2-prepared GE-EPI images (right column of panels). The effective echo times are similar for both images (30 ms for the conventional T2-weighted images; 28 ms for the T2-prepared GE-EPI images). Regions of interest (ROIs) of the tumor tissue are indicated with the red lines, showing absence of apparent geometric distortion within the tumor tissue in the EPI images. (TIF) [file pone.0099936.s003.tif]
