# Supplementary material for: Multiparametric MRI Analysis for the Identification of High Intensity Focused Ultrasound-Treated Tumor Tissue
Source: PLoS One. 2014 Jun 13;9(6):e99936. doi: 10.1371/journal.pone.0099936 (PMC4057317; doi:10.1371/journal.pone.0099936)
Supplement: Supporting Information S1 — Theory: the ISODATA clustering algorithm. (PDF) [file pone.0099936.s005.pdf]

Supporting Information S1
Theory: the ISODATA clustering algorithm

The ISODATA clustering described in the manuscript was performed with different feature vectors, consisting of either 1, 2, 3 or all 4 (T_1, T_2, ADC and MTR) MR contrast parameters. Extensive details of the ISODATA clustering algorithm can be found in the original paper by Jacobs et al. [1]. A brief theoretical description of the concepts of the clustering algorithm is given below.

If the clustering would be performed with a feature vector consisting of for example three parameters, the values in the tumor pixels can be represented in a 3D space, as schematically shown below, in which each black spot represents a single tumor pixel. To explain the ISODATA concept and for reasons of clarity, the clustering steps described in the figures below were performed on simulated data describing a limited number of well-separated clusters. The actual tumor data yielded typically 20-40 clusters, which would make the figures unclear.

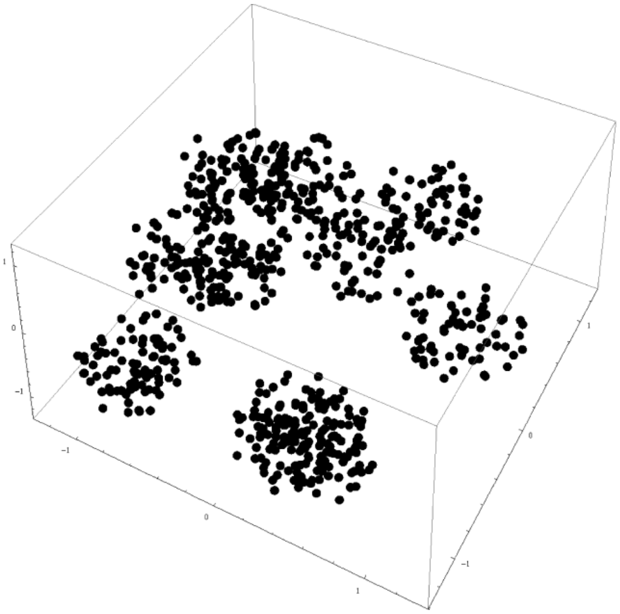

As the first step in the algorithm, an initial number of clusters is defined. In this example the initial number of clusters was set to 8. Cluster centroids (red spots below) are randomly defined for each cluster.

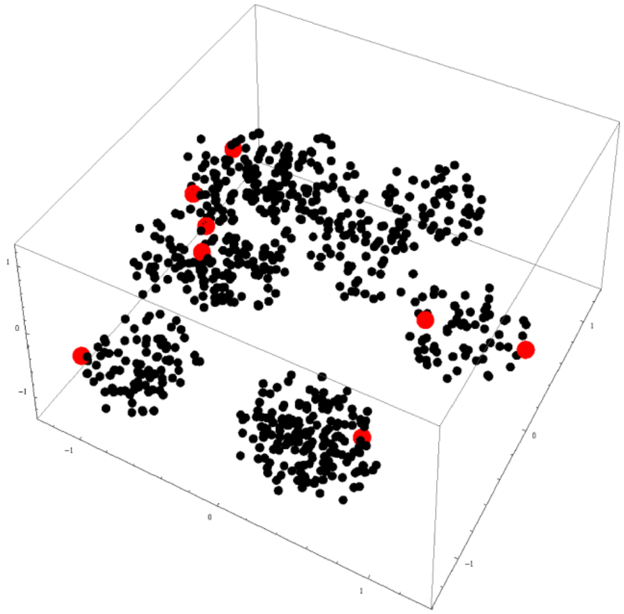

Next, pixels are assigned to the cluster of which the centroid is closest to the parameter values of the pixel. The different clusters that are the results of this step in the process are given by the different colors.

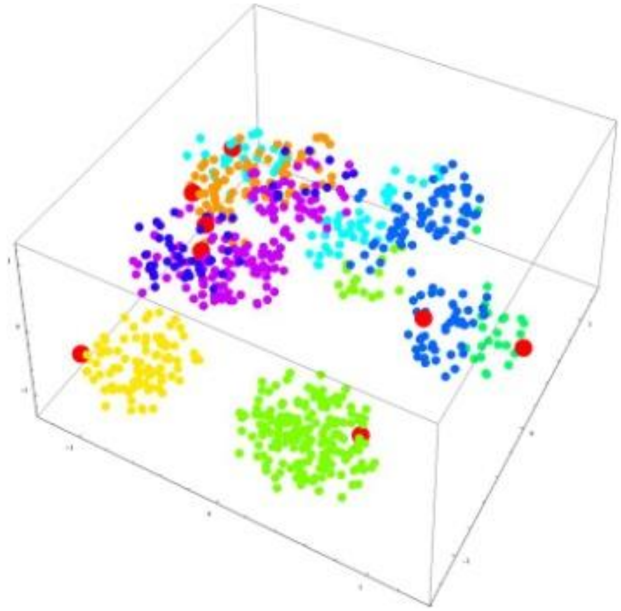

Subsequently, the centroids of each cluster are re-calculated. Clusters are split into two separate clusters, if the intra-Euclidean distances between the pixel vectors and the centroid of each cluster are larger than a pre-defined threshold. These intra-Euclidean distances are a measure of the spread within a certain cluster. Furthermore, pairs of clusters are merged into one cluster if the inter-Euclidean distance between the two cluster centroids is smaller than a pre-defined threshold, which means that the clusters are close to each other. In the example, the dark-blue and purple clusters were merged in this step, as can be seen below.

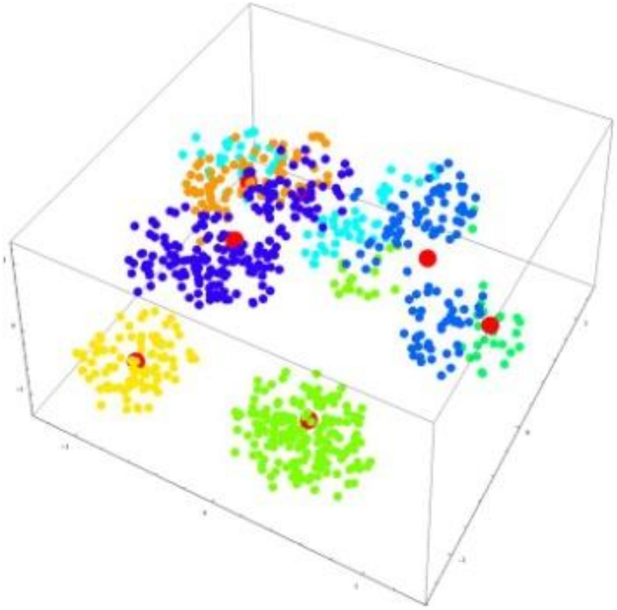

The previous step is repeated until convergence of the algorithm is reached. The algorithm is converged if the objective function [1] has not changed between subsequent iterations, which means that no alterations in the composition of the clusters occurred in the last iteration.

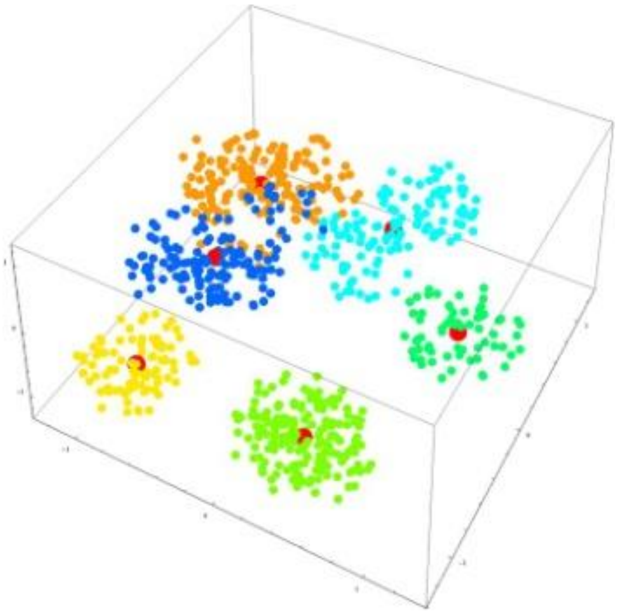

1. Jacobs MA, Knight RA, Soltanian-Zadeh H, Zheng ZG, Goussev AV, et al. (2000) Unsupervised segmentation of multiparameter MRI in experimental cerebral ischemia with comparison to T2, diffusion, and ADC MRI parameters and histopathological validation. J Magn Reson Imaging 11: 425-437.
